# Acoustic analysis of starting jets in an anechoic chamber: implications for volcano monitoring

**DOI:** 10.1038/s41598-020-69949-1

**Published:** 2020-08-11

**Authors:** J. J. Peña Fernández, V. Cigala, U. Kueppers, J. Sesterhenn

**Affiliations:** 1 Institut für Strömungsmechanik und Technische Akustik, Technische Universität Berlin, Berlin, Germany; 2 Department of Earth and Environmental Sciences, Ludwig–Maximilians–Universität (LMU), Munich, Germany; 3 Fakultät für Ingenieurwissenschaften, Universität Bayreuth, Bayreuth, Germany

**Keywords:** Volcanology, Fluid dynamics

## Abstract

Explosive volcanic eruptions are associated with a plethora of geophysical signals. Among them, acoustic signals provide ample information about eruptive dynamics and are widely used for monitoring purposes. However, a mechanistic correlation of monitoring signals, underlying source processes and reasons for short-term variations is incomplete. Scaled laboratory experiments can mimic a wide range of explosive volcanic eruption conditions. Here, starting (non-steady) compressible gas jets are created using a shock tube in an anechoic chamber and their acoustic signature is recorded with a microphone array. Noise sources are mapped in time and frequency using wavelet analysis and their dependence from pressure ratio, non-dimensional mass supply and exit-to-throat area ratio is deciphered. We observed that the pressure ratio controls the establishment of supersonic conditions and their duration, and influences the interaction between shock, shear layer, and vortex ring. The non-dimensional mass supply affects the duration of the discharge, the maximum velocity of the flow, and the existence of a trailing jet. Lower values of exit-to-throat area ratio induce a faster decay of the acoustic fingerprint of the jet flow. The simplistic experiments presented here, and their acoustic analysis will serve as an essential starting point to infer source conditions prior to and during impulsive volcanic eruptions.

## Introduction

Explosive volcanic eruptions are hazardous events. Large events from volcanoes with longer (years, decades, centuries) repose periods can to date be only poorly forecasted in terms of onset, eruption style and duration. A mechanistic correlation of monitoring signals, the underlying source processes and reasons for short-term variations is presently incomplete. Among the signals generated by erupting volcanoes and used for studying and monitoring purposes, we focus on the acoustic signature. Explosive volcanic eruptions produce a broad range of acoustic signals, from infrasound (< 20 Hz) to audible frequencies (20 Hz to 20 kHz). The dynamic range of this signal, over five orders of magnitude in its amplitude, allows deriving information on shallow processes based on frequency and wavelength variations. Moreover, and differently from seismic signals, which often represent both superficial and deep internal processes, acoustic signals provide a plethora of information about the near-vent eruptive dynamics.

Infrasound has been over the years the preferential frequency range because of its lower attenuation compared to that in the audible range, allowing successful measurements over distances of tens to hundreds of kilometers^1,2^. Infrasound signals have been used to infer key eruption parameters, such as exit velocity or mass ejection rate^3–5^, based on a theoretical and experimental investigation of the radiation of audible noise, stimulated by “episodic geyser-like eruptions of incandescent pyroclastics and gas” at Stromboli that produced noise “remarkably like that of twin jet engines”^6^. More quantitively, Taddeucci et al.^7^ investigated impulsive, seconds-long volcanic explosions and deciphered the sources and features of jet noise in the audible range. Goto et al.^8^ proposed a source of noise in the different frequency ranges. Specifically, they suggested that the infrasonic part of the signal is produced by magma doming at a free surface, while the high-frequency part reflects the consequent dynamics of gas and particle ejection.

## Background of the acoustics of supersonic jet flow and implications for volcanic processes

During explosive volcanic eruptions, magma is fragmented inside the conduit. Gas and pyroclast ejection dynamics into the atmosphere are the first direct observable. The interpretation of these dynamics requires knowledge of source (e.g. magma porosity, pressure) and path parameters (e.g. conduit geometry, roughness). Here, analysis of acoustic signals can provide constraints on these subsurface parameters.

Supersonic starting jet noise is produced by the impulsive release (volcanic explosion) of fluid contained in a pressurized reservoir at a pressure ratio large enough to deliver supersonic flow^9^. The noise of a single explosion is generated by several processes at various locations and the main components are^9^:

1. Compression wave generated by the sudden release of pressure;
2. Vortex ring noise, generated when the fluid rolls-up at the nozzle lip;
3. Turbulent jet mixing noise (TMN) caused by the turbulence in the jet;
4. Broadband shock noise (BBSN) induced by the interaction of shocks and the turbulence in the shear layer; and
5. Screech tone produced by the interaction between the shocks and the turbulence in the shear layer.

The difference of the present study is the temporal evolution of the noise sources in contrast to steady jet noise^10–12^. For non-steady jets as in the present study, a jet is composed of three phases: (1) the build-up phase leading to phase 2, (2) the quasi-steady jet phase, and (3) the final phase when the driving pressure is decaying.

**Figure 1:**
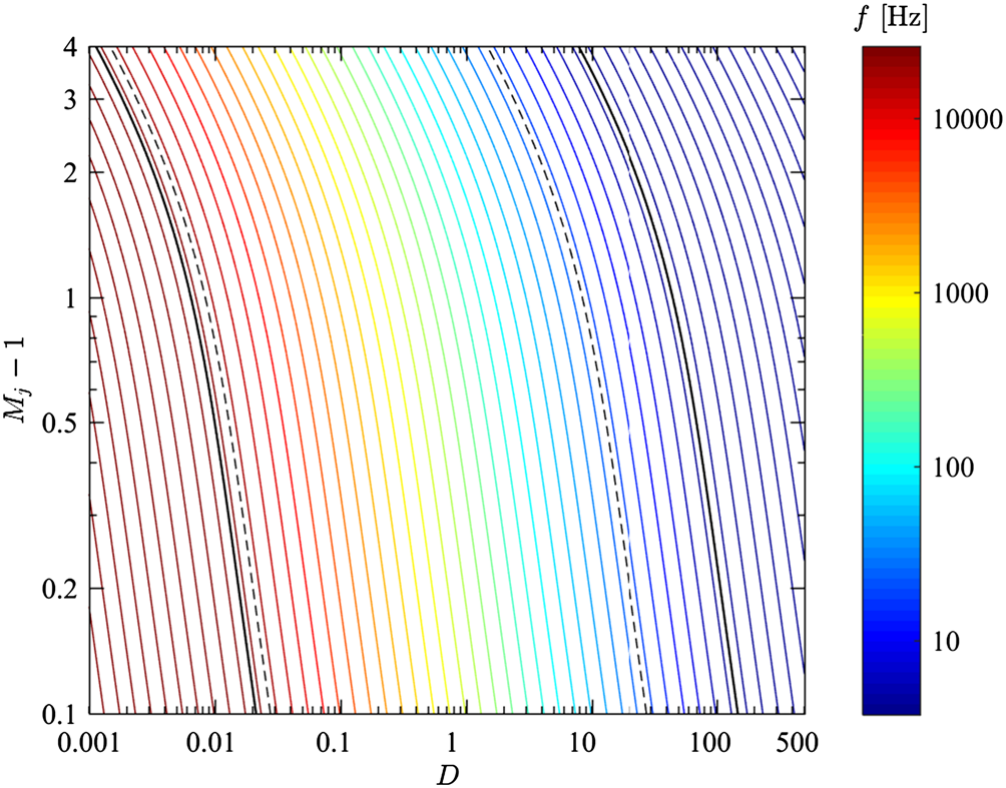
Peak frequency of the broadband shock noise (BBSN) radiated by a jet with different Mach numbers (M_j_) and diameters (D) for γ = 1.67 and a reservoir to unbounded chamber temperature ratio T_r_/T_∞_ = 1. The color-scale ranges from 3.75 to 20 000 Hz, the same frequency range as our microphone. This frequency range is also represented in the diagram with two solid black lines. The two dashed black lines correspond to the average human hearing frequency range (20–17 000 Hz).

1. The compression wave is a finite jump in pressure. For this reason, when performing a frequency analysis of the pressure time series, all frequencies are excited when the compression wave reaches the microphone. Compression waves travel at the speed of sound if they are generated with p_r_/p_∞_ lower than 41.2^13^. For larger values, it is considered a blast wave and its propagation Mach number is larger than 1. In general, larger p_r_/p_∞_ generate stronger compression waves but very large values are needed to generate compression waves with a noticeable larger amplitude^14^.
2. The vortex ring frequency can be estimated as proposed by Kopiev^15^ and Ran and Colonius^16^ using
  1 $$ f_{vr} = \frac{{u_{vr} }}{{\pi \mu^{2} D_{vr} \log \left( {\frac{8}{\mu } - \frac{1}{4}} \right)}} $$
  For symbol explanation see list of symbols.
3. The TMN is the dominant noise component in the downstream direction of the jet^9^ and generated by the turbulence of the jet flow. It cannot always be depicted as it is usually masked by the supersonic noise sources in the transverse direction. Both the large turbulent structures (also known as coherent structures and with a size of the order of the exit diameter D_e_) and the small vortices of the turbulence (also known as fine-scale turbulence and with a larger range towards smaller sizes when increasing the Reynolds number) contribute to the TMN although they are not radiated in the same direction. The TMN of large structures is radiated mainly downstream while the fine-scale TMN is radiated in the transverse direction. The fine-scale TMN peak frequency depends on the Reynolds number of the flow^17^ since the amount and size of large and small turbulent structures also depends on the Reynolds number, being the large turbulent structures always of a size similar to D_e_.
4. The broadband shock noise (BBSN) radiated by a jet depends on the shock-cell spacing L_s_ (distance between two shocks) and the propagation velocity of the Kelvin–Helmholtz vortices (assuming u_KH_ = 0.7 u_j_). The related peak Strouhal number is 1^18^:
  2 $$ \frac{{f_{BBSN} L_{s} }}{{u_{KH} }} = 1 $$
  and describes a universal behavior for supersonic jets. The shock-cell spacing depends on the throat diameter, the Mach number, and the isentropic exponent of the gas in the reservoir (see equations below). The eddy convection velocity depends mainly on the Mach number and the isentropic exponent of the gas in the reservoir. At volcanoes, geometry and gas compositions vary. Unfolding the dependence of the shock-cell spacing and the eddy convection velocity with the Mach number and exit diameter, we show the peak frequency of the BBSN for different Mach numbers and exit diameters (Fig. 1). Experimental setups (millimeter to centimeter throats) produce a jet which radiates BBSN frequencies above 10 kHz over a large range of Mach numbers, while natural volcanic jets (released from decimeter- to meter-sized vents that are on top of conduits of so far unconstrained dimensions) produce jets that radiate in the range of 20–1,000 Hz. For even larger diameters, e.g. 100 m, the BBSN frequencies are in the infrasonic range (down to 0.1 Hz or even 0.01 Hz)^19^. Based on the scaling law of Peña Fernández^9^, the peak frequencies of the supersonic jet noise sources of laboratory experiments can be scaled to nature without losing accuracy:
  3 $$ f_{BBSN} = \frac{{\sigma_{1} }}{{\pi \sqrt {M_{j}^{2} - 1} }}\frac{{c_{j} }}{{D_{j} }} $$
  where the different parameters can be calculated using:
  3.1 $$ D_{j} = D_{st} \left( {\frac{{1 + \frac{1}{2}\left( {\gamma - 1} \right)M_{j}^{2} }}{{1 + \frac{1}{2}\left( {\gamma - 1} \right)M_{d}^{2} }}} \right)^{{\frac{\gamma + 1}{{4\left( {\gamma - 1} \right)}}}} \left( {\frac{{M_{d} }}{{M_{j} }}} \right)^{\frac{1}{2}} $$
  3.2 $$ M_{j} = \left( {\frac{2}{\gamma - 1}\left( {\left( {\frac{{p_{0r} }}{{p_{\infty } }}} \right)^{{\frac{\gamma - 1}{\gamma }}} - 1} \right)} \right)^{\frac{1}{2}} $$
  3.3 $$ \frac{{A_{e} }}{{A^{*} }} = \left( {\frac{2}{\gamma + 1}} \right)^{{\frac{\gamma + 1}{{2\left( {\gamma - 1} \right)}}}} \frac{1}{{M_{d} }}\left( {1 + \left( {\frac{\gamma - 1}{2}M_{d}^{2} } \right)} \right)^{{\frac{\gamma + 1}{{2\left( {\gamma - 1} \right)}}}} $$
  3.4 $$ c_{j} = \sqrt {\gamma R_{g} T_{j} } $$
  3.5 $$ T_{j} = T_{r} \left( {1 + \frac{\gamma - 1}{2}M_{j}^{2} } \right)^{ - 1} $$
  For symbols’ explanation see list of symbols.
5. The scaling law for the screech tone frequency according to Schulze^20^ is:
  4 $$ f_{screech} = \frac{{0.67M_{j} c_{j} D_{j} }}{{\sqrt {M_{j}^{2} - 1} }}\left( {1 + \frac{{0.7M_{j} }}{{\sqrt {\left( {1 + \frac{\gamma - 1}{2}M_{j}^{2} } \right)} }}\sqrt {\frac{{T_{\infty } }}{{T_{r} }}} } \right)^{ - 1} $$

For symbols’ explanation see list of symbols.

The behavior of a starting jet with a fixed geometry is governed by four main dimensionless parameters^21^: (1) the Reynolds number (Re), affecting mainly the size of the Kelvin–Helmholtz instabilities relative to the nozzle diameter,(2) the non-dimensional mass supply (L/D_st_) given by the length (L) of a hypothetical cylindrical reservoir of diameter D_st_, influencing the existence of a trailing jet as well as the maximum velocity of the flow at the nozzle exit; (3) the reservoir-to-unbounded chamber pressure ratio (p_r_/p_∞_), having an effect on the compressibility and (4) the reservoir-to-unbounded chamber temperature ratio T_r_/T_∞_, also having an effect on compressibility.

Another important non-dimensional parameter of jet dynamics is the characteristic time (t^*^ = t D_st_/u_j_), defined as the time the flow needs to advance the distance of one nozzle diameter at the typical velocity of the flow. For instances, using the cylindrical nozzle with 28 mm diameter, one characteristic time is 0.04 ms.

**Figure 2:**
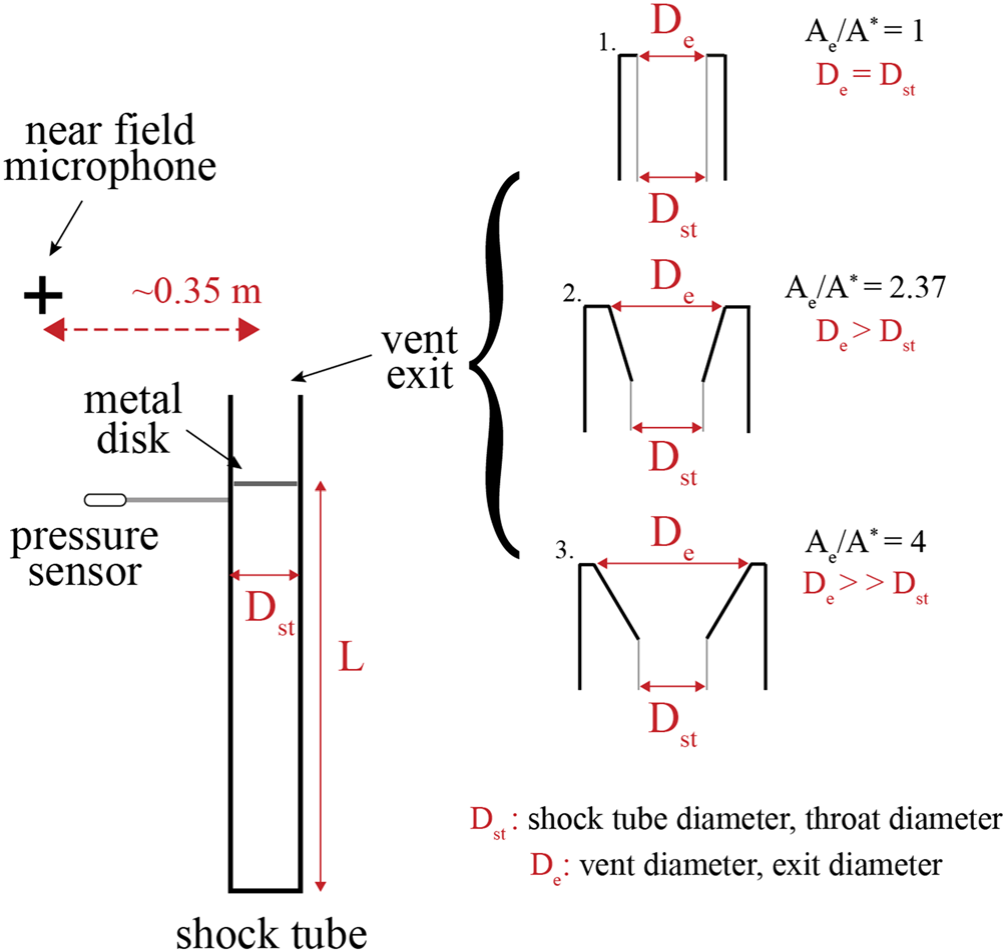
Setup configuration. Shock tube with a constant diameter D_st_ and variable length L. Three shock-tube exit geometries were studied as represented: (1) straight, (2) slightly divergent and (3) strongly divergent. The microphone was located at a similar height to the shock-tube exit at a distance of 12.5 D at an angle of 2.29°. In the setup used, D_st_ also represents the throat diameter, the smallest diameter the flow as to pass through. D_e_ is the exit diameter and it also represents the vent diameter.

In this work, we focus on acoustic signals in the audible frequency range. We produced starting compressible gas jets under controlled conditions in the laboratory using a shock tube apparatus^22,23^ and recorded the acoustic signature produced by the impulsive decompression in the anechoic chamber (Fig. 2) and mapped noise sources in time and frequency. The complete dataset with starting conditions and raw acoustic measurements is available through the EPOS database^24^. This will be the foundation for future quantitative analysis of experiments with increased geometrical complexity, elevated reservoir temperature and containing particles and will ultimately serve to scale the measurable noise of volcanic eruptions to the underlying conditions. Varying some governing parameters (pressure and geometry) allowed to empirically constrain their influence on acoustic properties of the generated jets.

With reservoir temperature and gas species constant, we varied the following parameters (Fig. 2) (and list the volcanic parameter in parentheses):

- Reservoir-to-unbounded chamber pressure ratio (p_r_/p_∞_) = [3, 4, 50 and 80]. (Gas overpressure inside gas bubbles in magma)
- non-dimensional mass supply (L/D_st_) = [2, 8] (gas bubble or slug size)
- nozzle geometry parametrized by the exit-to-throat area ratio (A_e_/A*) = [1, 2.37, 4] (conduit and vent geometry)

## Results

### Acoustic fingerprint identification of the physical elements in the wavelet diagram

**Figure 3:**
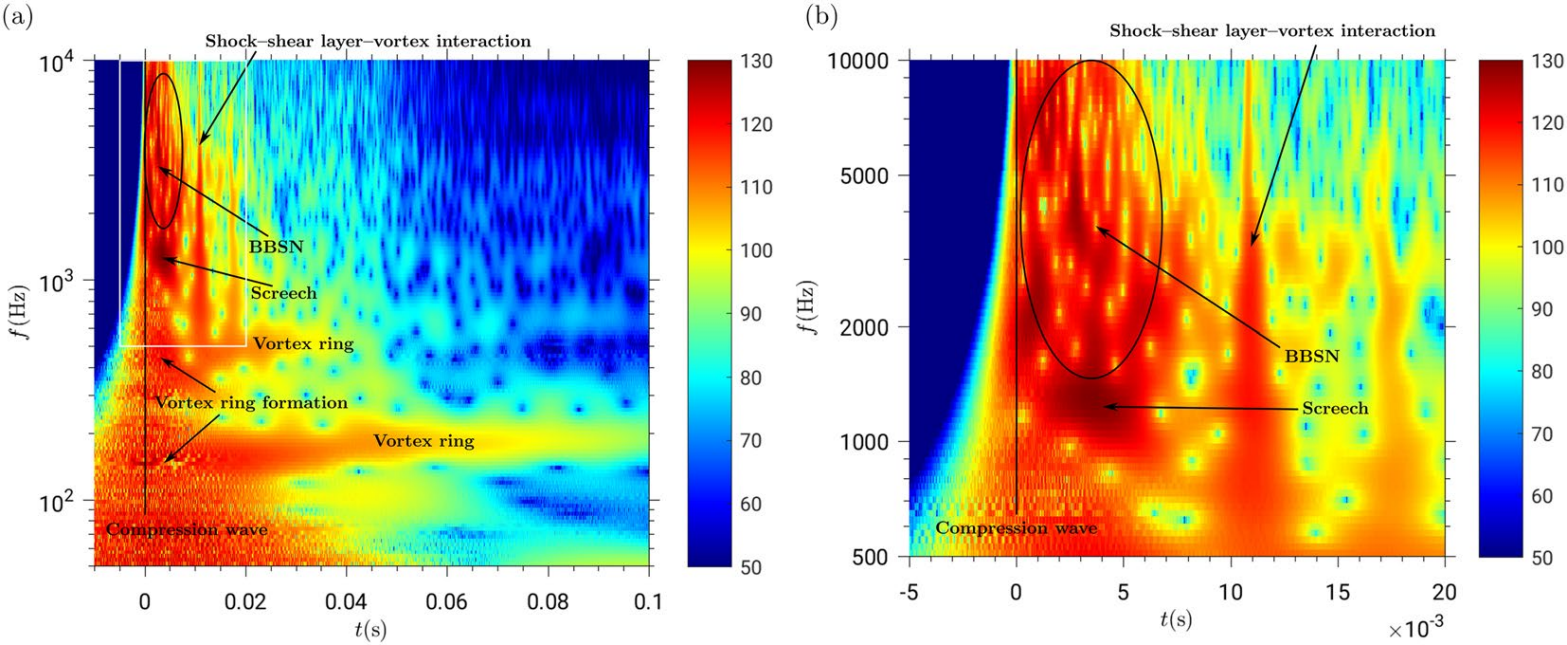
Identification of the elements of the starting jet in the wavelet diagram. The color scale represents the sound pressure level [dB] at the microphone location. Pressure ratio p_r_/p_∞_ = 50; L/D = 8; A_e_/A^*^ = 1. (**a**) The compression wave, vortex ring, screech, broadband shock noise and the shock-shear layer-vortex interaction can be identified in the diagram by matching the frequency and time delay estimations with the patterns found in the spectrogram from the recorded signals. The white frame is the region represented in panel (**b**). (**b**) Zoom in of the wavelet diagram. BBSN and screech can be identified as well as the shock-shear layer-vortex interaction.

To locate the acoustic components in frequency and time, we transformed the signal into the frequency space using the wavelet decomposition (see section “Methods” for details). In this way, we can identify the acoustic fingerprint of every element of the compressible starting gas jet. Figure serves as an example for experiments at p_r_/p_∞_ = 50, L/D_st_ = 8, A_e_/A* = 1 and the acoustic fingerprints are presented in the following:

- At the gas release time (t = 0), the fingerprint of the compression wave can be identified by the excitation of all frequencies. Note here that in the wavelet transform the temporal resolution is lower for lower frequencies, which leads to the broadening of the signal in time for lower frequencies^25^.
- When the contact surface of the flow (the boundary between the fluid located initially inside and outside the reservoir) reaches the exit of the shock tube, a vortex ring is formed, radiating a frequency of 2.45 kHz. With time, a vortex ring grows radially while decelerating vertically^26^, leading to an almost constant frequency radiated over time. For the set of parameters in this study, this frequency was in the range of 200 Hz.
- About ten characteristic times after release (0.4 ms), a jet flow is fully developed at the shock tube exit, as identified in the wavelet diagram by the relatively large amplitude (approximately 120 dB for the setup in this study). For different frequency regimes, we can identify the different components of jet noise, when measuring the acoustic signal at a position close to the shock tube exit plane (see measurement position in Fig. 2).
- The BBSN peaks at 3.51 kHz in Fig. 3^,^ as expected following Peña Fernández^9^.
- The screech can be found at a frequency of 1.4 kHz in Fig. 3^,^ which agrees with Schulze^20^. However, as the analyzed jets are not steady, the screech frequency decreases with time to reach a minimum frequency (around 1 kHz), followed by another rise as the reservoir discharges. This frequency shift is due to the (non-linear) dependence with the Mach number^9^. Usually, the screech tone has a similar frequency to the lowest frequency radiated by the BBSN^27^. This pattern becomes obvious in Fig. 3b, where the lowest part of the dark red spot (t = 6 ms and frequency around 1 kHz) corresponds to the screech tone while the adjacent dark red zone with slightly larger frequencies (not separated by a low amplitude region) corresponds to the BBSN.
- The peak frequency of the fine-scale TMN depends on the Reynolds number^17^. The range of Reynolds numbers present in this study (around 10^7^) leads to a peak frequency for the fine-scale TMN around 10 kHz, corresponding to the range of the BBSN and therefore masked by it.
- Acoustic fingerprints similar to the one of the compression wave are present in the wavelet diagram of Figure b for t = 11 ms. This secondary acoustic wave is due to the interaction between the shock, the shear layer and the vortex ring as first reported by Peña Fernández and Sesterhenn^21^. The acoustic wave resulting from this interaction was observed, as expected by^21^, 11 ms after the compression wave (see Fig. 3b “shock—shear layer—vortex ring interaction”). This interaction has been identified for the first time in an experimental acoustic dataset. These secondary acoustic waves cannot correspond to secondary pressure waves due to reflections at the bottom of the reservoir: for L/D_st_ = 8, the delay with respect to the primary compression wave is in the order of 1 ms; for L/D_st_ = 2, the delay is in the order of 0.1 ms. This signature can furthermore not correspond to reflections at the walls of the anechoic chamber because the expected delay with respect to the compression wave would be 20 ms (based on the size of the anechoic chamber and the position of the setup).

### Effect of the main parameters on the acoustic fingerprint

In our measurements, we have varied the pressure ratio (p_r_/p_∞_), the non-dimensional mass supply (L/D_st_) and the exit-to-throat area ratio (A_e_/A^*^) and show their effect on the acoustic fingerprint changes.

**Figure 4:**
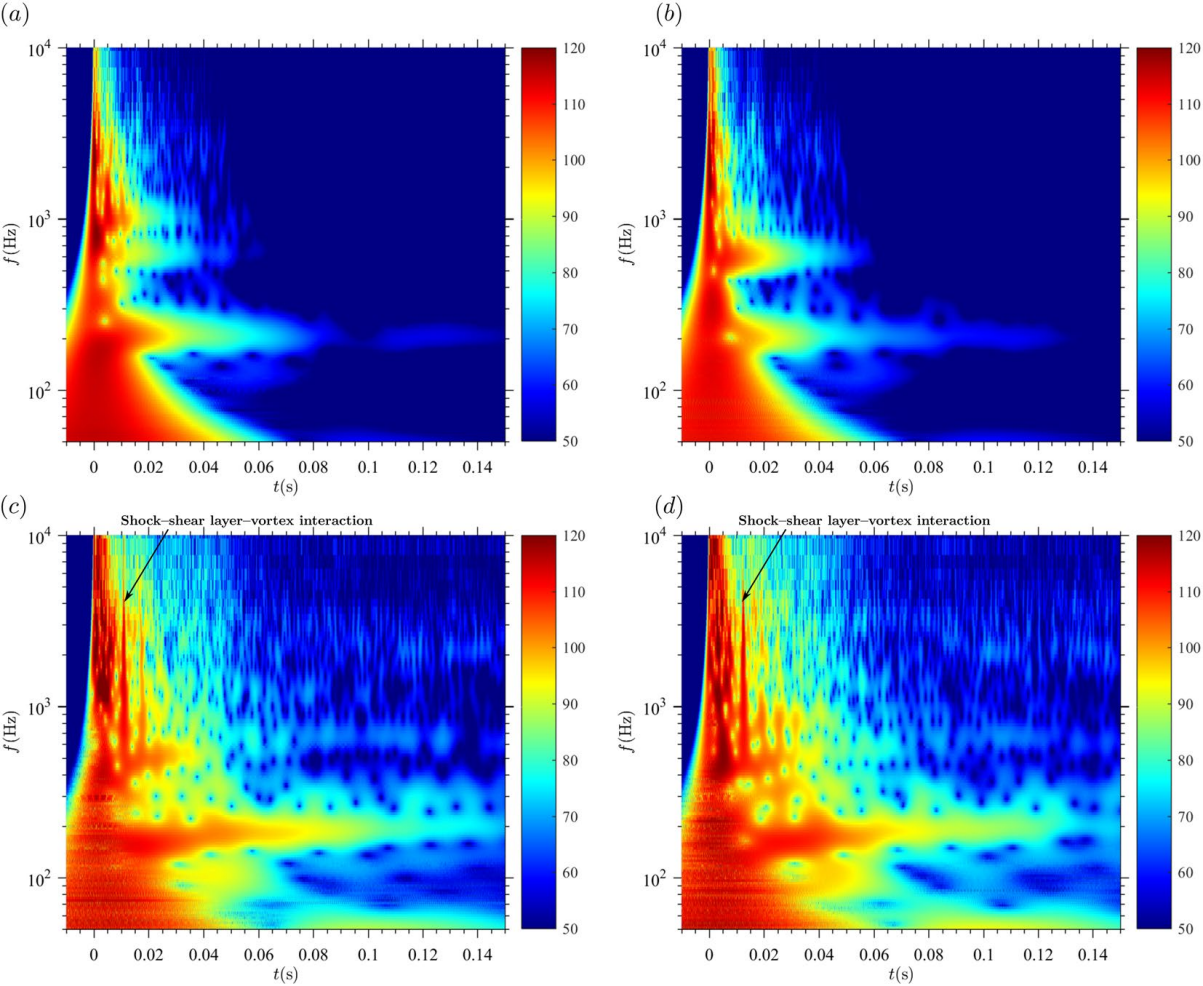
Effect of the pressure ratio on the acoustic signature of the shock-tube in the wavelet diagram. The color scale represents the sound pressure level [dB] at the microphone location. Ae/A* = 1 and L/D = 8. (**a**) p_r_/p_∞_ = 3. (**b**) p_r_/p_∞_ = 4. (**c**) p_r_/p_∞_ = 50. (**d**) p_r_/p_∞_ = 80.

**Figure 5:**
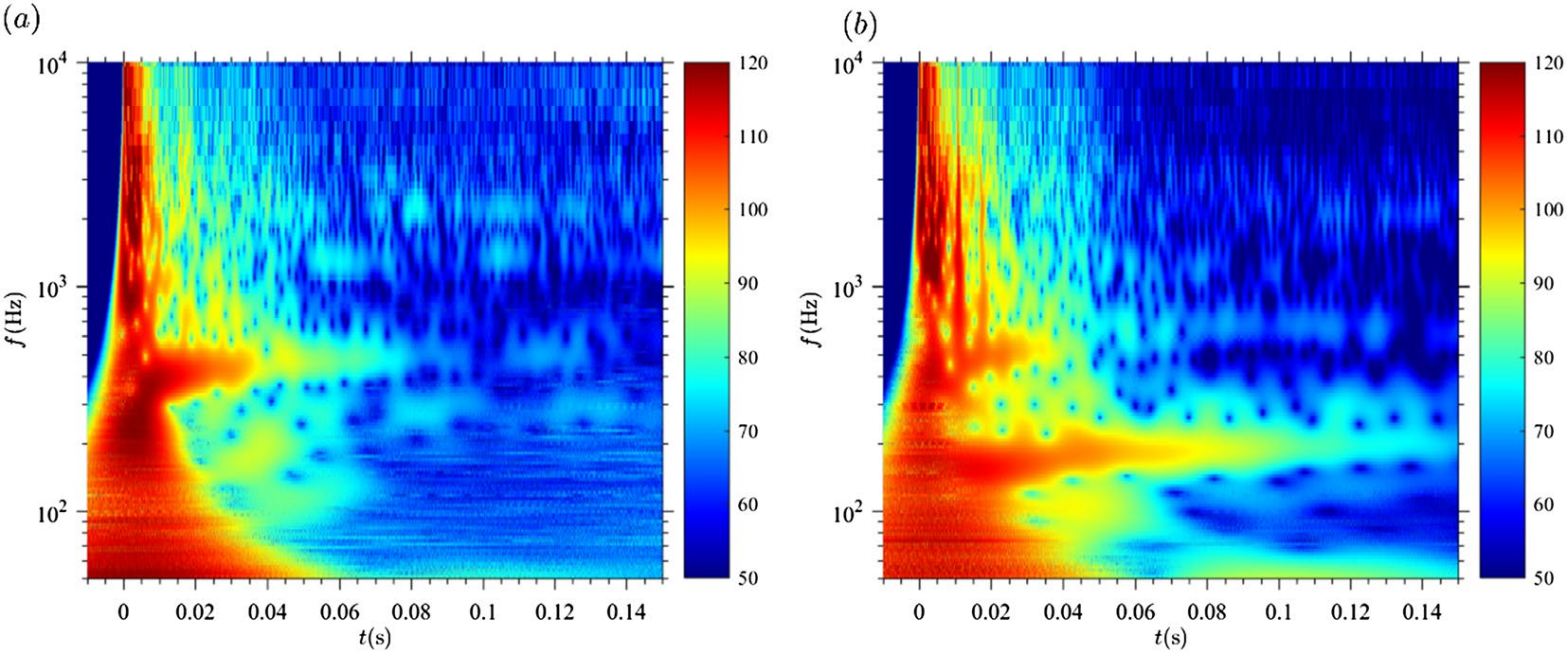
Effect of the non-dimensional mass supply on the acoustic signature of the shock-tube in the wavelet diagram. The color scale represents the sound pressure level [dB] at the microphone location. A_e_/A* = 1 and p_0r_/p_∞_ = 50. (**a**) L/D = 2. (**b**) L/D = 8.

1. Pressure ratio (Fig. 4)
2. Concerning the compression wave, we observed no variation of frequency or amplitude on the recorded signals, as all frequencies have been excited for all experiments in this study and the arrival of the compression wave at the microphone was used to synchronize our experiments.
3. Regarding the vortex ring, its frequency is approximately constant (around 200 Hz; although slightly larger for p_r_/p_∞_ = 3 and 4, as expected from Kopiev^15^. On the other hand, its amplitude and duration change noticeably and are positively correlated with pressure ratio, because the amount of energy and vorticity contained in the vortex is larger. for p_r_/p_∞_ = 3, the fingerprint vanishes after approximately 0.09 s. For p_r_/p_∞_ = 4, the acoustic fingerprint vanishes after approximately 0.13 s while for p_r_/p_∞_ = 50 and above, the acoustic fingerprint is even longer, and the end is not represented in Fig. 4. Amplitude and vortex ring noise are also positively correlated with pressure ratio: at t = 0.05 s for example, the amplitude for p_r_/p_∞_ = 50 is around 100 dB while around 70 dB for p_r_/p_∞_ = 3 and 4.
4. No BBSN nor screech tone were recorded for p_r_/p_∞_ = 3 and 4.
5. TMN is the dominant noise component (which can be seen just after the compression wave), ranging in frequency from 600 to 10,000 Hz. It evolves towards lower values because the vortices grow (a size more similar to that of the shock tube exit) as the Reynolds number of the flow decreases. For p_r_/p_∞_ = 50 and 80, the supersonic jet noise components (BBSN and screech) are dominant and partially mask the TMN. Screech has a lower frequency for p_r_/p_∞_ = 80 (about 450 Hz) than for p_r_/p_∞_ = 50 (about 1200 Hz). After the supersonic jet noise sources disappear, TMN still radiates some noise, but at diminishing rates. For p_r_/p_∞_ = 80 the decay is slower than for p_r_/p_∞_ = 50.
6. Both high-pressure cases show the shock-shear layer-vortex interaction, although not for the same delay with respect to the compression wave. The low-pressure cases do not show any interaction between the shock-wave, the shear layer and the vortex ring because the supersonic phase, if existent, has duration too short for such interaction to take place.
7. This delay depends on the shock-cell spacing (L_s_), which is, in turn, mainly dependent on the pressure ratio, and the velocity of the Kelvin–Helmholtz instabilities of the jet flow. We found that the delay can be calculated as follow:
  5 $$ dt_{s - sl - vr} = \frac{{L_{s} }}{{u_{KH} }} $$
  Using the following relationships:
  5.1 $$ \frac{{L_{s} }}{{D_{st} }} = \frac{\pi }{{\sigma_{1} }}\sqrt {M_{j}^{2} - 1} \frac{{D_{j} }}{{D_{st} }} $$
  5.2 $$ \frac{{D_{j} }}{{D_{st} }} = \left( {\frac{{1 + \frac{\gamma - 1}{2}M_{j}^{2} }}{{1 + \frac{\gamma - 1}{2}M_{d}^{2} }}} \right)^{{\frac{\gamma + 1}{{4\left( {\gamma - 1} \right)}}}} \left( {\frac{{M_{d} }}{{M_{j} }}} \right)^{\frac{1}{2}} $$
  5.3 $$ \frac{{p_{r} }}{{p_{\infty } }} = \left( {1 + \frac{\gamma - 1}{2}M_{j}^{2} } \right)^{{\frac{\gamma }{\gamma - 1}}} $$
  5.4 $$ u_{KH} = 0.7 u_{j} = 0.7M_{j} c_{j} $$
  5.5 $$ \frac{{c_{0} }}{{c_{j} }} = \left( {1 + \frac{\gamma - 1}{2}M_{j}^{2} } \right)^{\frac{1}{2}} $$
  where we assume the velocity of the Kelvin–Helmholtz instabilities to be 70% of the fully expanded velocity.
  We get to:
  6 $$ dt_{s - sl - vr} \sim \frac{{\left( {M_{j} } \right)^{{\frac{1}{\gamma - 1}}} }}{{M_{j} }}\sim \frac{{\left( {\frac{{p_{r} }}{{p_{\infty } }}} \right)^{\frac{1}{2}} }}{{\left( {\frac{{p_{r} }}{{p_{\infty } }}} \right)^{{\frac{\gamma - 1}{{2\gamma }}}} }}\sim \left( {\frac{{p_{r} }}{{p_{\infty } }}} \right)^{{\frac{1}{2\gamma }}} $$
  which means for Argon (γ = 1.67) an exponent of 0.299. The average value of the exponent in the cases here presented is 0.3 and the exponents of all cases are within the interval (0.28, 0.32), which agrees very well with the analytical solution.
8. Non-dimensional mass supply (L/D_st_) (Fig. 5)
  The non-dimensional mass supply (L/D_st_) has three main effects: (i) the duration of the discharge and (ii) the maximum velocity of the flow are positively correlated with L/D_st_ and (iii) the existence of a trailing jet is expected for values of L/D_st_ larger than approximately four^28^. Figure 5 shows the acoustic fingerprint of A_e_/A^*^ = 1 and p_r_/p_∞_ = 50. L/D_st_ varied between 2 and 8.
9. Discharge duration and maximum flow velocity are larger for L/D_st_ = 8 than for L/D_st_ = 2. For t = 0.05 s, the noise radiated by L/D_st_ = 8 is significantly larger than for L/D_st_ = 2.
10. The shock-shear layer-vortex interaction can be observed for L/D_st_ = 8 but not for L/D_st_ = 2.
11. The vortex ring can be identified for both cases around 600 Hz. For L/D_st_ = 8, an additional vortex ring with a lower frequency (around 200 Hz) can be observed. This additional vortex ring propagates at a lower velocity.
12. Nozzle geometry, parametrized by the exit-to-throat area ratio (Figure)

**Figure 6:**
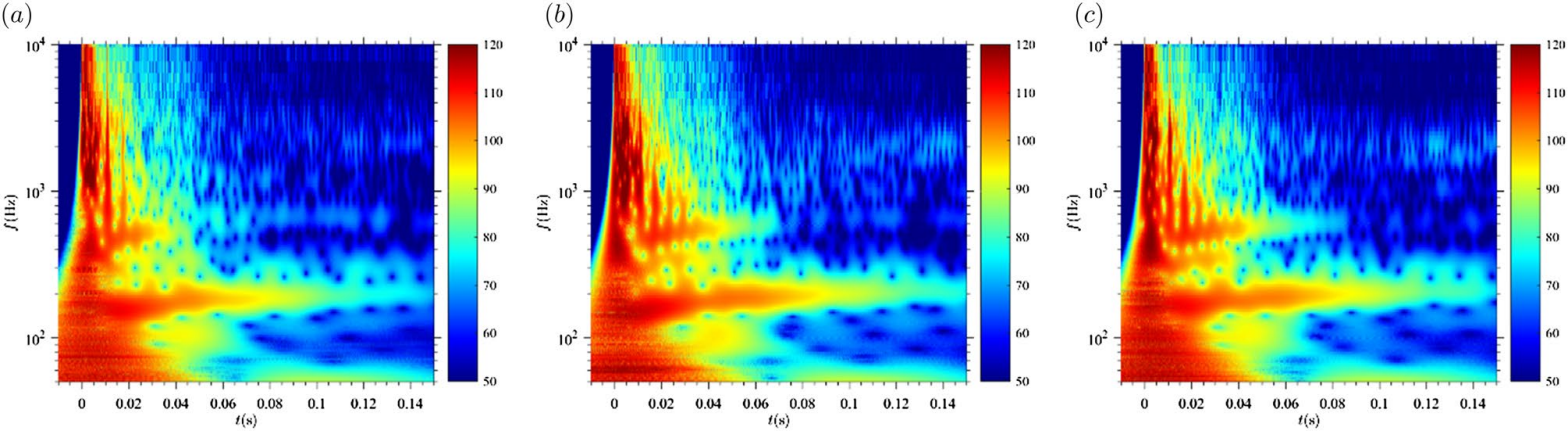
Effect of the nozzle geometry on the acoustic signature of the shock-tube in the wavelet diagram. The color scale represents the sound pressure level [dB] at the microphone location. L/D = 8 and p_0r_/p_∞_ = 50. (**a**) A_e_/A^*^ = 1. (**b**) A_e_/A^*^ = 2.36. (**c**) A_e_/A^*^ = 4.

The nozzle geometry affects the portion of the flow inside the setup before crossing the nozzle exit plane. A divergent nozzle enables the flow to expand in the supersonic regime but causes compression in the subsonic regime. The larger A_e_/A*, the stronger this effect. Figure 6 shows the acoustic fingerprint of L/D_st_ = 8 and p_r_/p_∞_ = 50. A_e_/A* varied from 1 to 4.

- Frequency of compression wave and vortex ring does not change. However, larger A_e_/A* generates more stable vortex rings with accordingly larger amplitudes for a longer time.
- The decay of the acoustic fingerprint of the jet flow (i.e. TMN, BBSN and screech) is negatively correlated (see the time evolution of the three cases presented in Fig. 6 at a frequency around 600 Hz). Once the flow at the throat is no longer choked (= subsonic), the flow velocity will be higher for A_e_/A^*^ = 1 as the divergent geometry acts as a diffuser. The delay of the shock-shear layer-vortex ring interaction with respect to the compression wave is constant (11 ms).
  7 $$ dt_{s - sl - vr} = \frac{{L_{s} }}{{u_{KH} }} $$

Using the following relationship (and 5.1 to 5.5):

$$ u_{KH} = 0.7M_{d} c_{e} $$

where the velocity of the Kelvin–Helmholtz instabilities is written as a function of the conditions at the nozzle exit. For symbols’ explanation see list of symbols.

We get to:

8 $$ dt_{s - sl - vr} \sim \frac{{M_{d}^{{\left( {\frac{1}{2} \frac{\gamma + 1}{{4\left( {\gamma - 1} \right)}} + \frac{1}{2}} \right)}} }}{{M_{d} }}\sim M_{d}^{{\frac{5 - 3\gamma }{{8\left( {\gamma - 1} \right)}}}} \sim \left( {\frac{{A_{e} }}{{A^{*} }}} \right)^{{\frac{5 - 3\gamma }{{16}}}} $$

where the exponent takes the value of − 6.25·10^–4^ for γ = 1.67.

## Discussion

Among the signals generated by volcanoes and used for monitoring purposes, we chose the acoustic signal as it provides information about the eruptive dynamics in the near-vent region and allows for correlating parameters of particle ejection and eruption column to the underlying (mostly subsurface) conditions (e.g., geometry, pressure) via empirical relationships. However, most of the state-of-the-art correlations are based on simple geometrical systems, and the complex volcanic features are often not taken into account as underlined by Matoza et al.^3,11^. Swanson et al.^29^ were the first performing an experimental investigation of jet acoustic issuing from nozzles of different geometries with application to volcanoes. They used nozzles with convergent, cylindrical, and divergent walls (A_e_/A^*^ ranging from 0.1 to 2). The experimental setup is, in general, different from the one we used, and they did not minimize external noise in their runs. They were able to distinguish jet noise from structural features of flows exiting the convergent nozzles only. We performed acoustic measurements of a shock tube in an anechoic chamber varying the pressure ratio (p_r_/p_∞_), the non-dimensional mass supply (L/D_st_) and the exit-to-throat area ratio (A_e_/A^*^). Performing the experiments in an anechoic chamber gave us the possibility to define the signature of all the different sources of noise with high accuracy and map them in time and frequency. Based on the timing and frequency estimations of the different elements of the starting jets, we have been able to identify multiple elements in the wavelet diagram and the controlling parameters:

The compression wave is a jump in pressure, and when transformed to the frequency space, it can be identified by the excitation of all frequencies for a single time. No variation is expected in the frequency distribution of the compression wave since all frequencies will be excited. This element is present in all cases that we have measured. We expected to have a slightly stronger compression wave for larger p_r_/p_∞_, which is represented by larger amplitudes in the acoustic fingerprint but the values used in this study do not lead to large differences in the noise amplitude of the compression wave. Noticeable larger p_r_/p_∞_ are needed to perform a proper analysis. We synchronized all recorded signals using the arrival of the compression wave to the microphone; hence we have no information about the propagation velocity of the compression wave in the different cases.

The vortex ring is generated at the lip of the shock tube exit when the flow rolls up due to the instability caused by the large velocity gradients. Vortex rings are very robust structures that can travel long distances without becoming unstable^26^. While propagating in the axial direction, the vortex ring radius grows, and the propagation velocity decreases. For this reason and according to Eq. (1), the frequency radiated by the vortex ring is almost constant over time. We have confirmed that for larger p_r_/p_∞_, the vortex ring frequency decreases slightly. For L/D_st_ = 8, an additional vortex ring was observable in the wavelet diagram with a lower frequency, meaning that it moves much slower than the first one (around one third of the velocity assuming a similar shape than the former vortex ring), driven by generally lower exit velocities with time. This also agrees with the accepted theory of Gharib^28^ that the trailing jet exists for L/D_st_ > 4. Larger values of A_e_/A^*^ are positively correlated with stability and noise radiation duration of the vortex rings. We observed vortex rings under all experimental parameter combinations. Quantifying vortex ring characteristics of explosive eruptions (diameter and its evolution, propagation velocity) will contribute to constraining the commonly unknown vent diameter^26^.

The quasi-steady trailing jet has established a few characteristic times after the release of the pressure from the shock tube for L/D_st_ = 8. In this case, supersonic noise sources are dominant over subsonic ones^27^.

In the wavelet diagram for the supersonic cases, we identified the BBSN as a high amplitude region. Its frequency can be estimated using equation (). Its frequency decreases for increasing Mach numbers and therefore for larger p_r_/p_∞_ and L/D_st_. The duration of the quasi-steady jet phase can be correlated with L/D_st_. Assuming a pressure ratio large enough to deliver sonic flow at the throat and a known vent diameter (see equations in the sections above), this correlation will give insights into erupted gas volume (and possibly fragmentation depth), but this topic needs further investigation.

The screech tone showed the highest amplitude of noise sources. Its frequency is lower than that of the BBSN and was found to be negatively correlated with Mach number. Accordingly, its frequency can be estimated using equation ().

Following Bailly and Bogey^17^ and for a Reynolds number of 10^7^, the TMN peak frequency is about 10 kHz for the experiments in this study. As this frequency is similar to that of the BBSN, this noise source is masked by BBSN and therefore does not provide any additional information.

For some combinations of p_r_/p_∞_ and L/D_st_, we did not identify any supersonic jet noise source (BBSN, TMN, screech) in the wavelet diagrams, meaning that the jet had never reached supersonic conditions. We speculate that, if generated at all, the duration of supersonic flow was too short to radiate supersonic jet noise components, i.e. insufficient time for the vortices of the flow to interact with the shock waves.

We could identify an acoustic wave resulting from the interaction between the shock, the shear layer and the vortex ring, theoretically first postulated by Peña Fernández and Sesterhenn^21^. The delay between compression and acoustic wave is independent of A_e_/A^*^ and depends on p_r_/p_∞_ (see Eqs. 6 and 8). This could be quantified remarkably well for the different experimental conditions of this study (Figs. 4c,d, 5b and 6).

The results obtained in this study can be used to attempt upscaling of the experimentally constrained relationships to volcanic systems. The equations presented above show that there are relationships between Mach number, gas properties, A_e_/A^*^, p_r_/p_∞_, L/D_st_ and the peak frequency of the supersonic jet noise sources. Taking BBSN as an example, Fig. 1 shows that its dominant frequency is negatively correlated with D_e_. Based on Eq. 3, the peak frequency of the BBSN would be approximately 170 Hz when assuming (i) a cylindrical conduit and vent of 1 m diameter, (ii) gas composition (80% water, 10% CO_2_ and 10% SO_2_) leading to an isentropic exponent of γ = 1.31 and a gas constant R_g_ = 401.1, and (iii) a Mach number M = 3. For reference, in the experimental setup with a conduit diameter of 28 mm and a composition 100% Ar (γ = 1.67 and R_g_ = 208) at a Mach number of M = 3, the peak frequency of the BBSN is 3.51 kHz.

We are aware that volcanic jets are characterized by even more complex dynamics than the ones here tested (e.g. presence of particles, rough conduit, dynamic evolution of the vent area, external noise, etc.). However, we can characterize all the important elements of the shock tube discharge from acoustic measurements using a single microphone: compression wave, vortex ring, the components of the supersonic jet noise and the interaction between the shock, the shear layer and the vortex ring. At low particle concentration in a dilute flow, robust supersonic structures may not be significantly affected. We refer to the investigation of further complexities, like the presence of particles, but also less clean ambient conditions to future work. “Clean” laboratory experiments in an anechoic room are an essential starting point for future links between scaled and reproducible laboratory experiments (with all starting parameters known) and natural volcanic eruptions (without any parameter known a priori).

## Conclusions

The analysis of acoustic signals is nowadays widely used for volcano monitoring purposes. Nevertheless, many uncertainties remain in the parameters retrieved from acoustic analysis due to the complexity of volcanic systems. The present investigation focused on a better understanding of the relationship between source parameters and acoustic signals generated by compressible starting gas jets. Compressible starting (i.e. non-steady) gas jets have been generated using a shock tube system in an anechoic chamber and their acoustic noise sources have been located in time and frequency using wavelet analysis. The effect of the following parameters has been analyzed: pressure ratio (p_r_/p_∞_), non-dimensional mass supply (L/D_st_) and exit-to-throat area ratio (A_e_/A^*^).

- p_r_/p_∞_ controls (1) the establishment of supersonic conditions in the jet flow, (2) the duration of supersonic conditions in the jet flow, and (3) the interaction between shock, the shear layer, and the vortex ring.
- L/D_st_ is positively correlated with (1) the duration of the discharge, and (2) the maximum flow velocity and affects (3) the existence of a trailing jet.
- A_e_/A^*^ is negatively correlated with the duration during which individual acoustic fingerprints can be observed.

We have performed this study under controlled ambient conditions in the anechoic chamber, which gave us the possibility to focus on the acoustic fingerprints of each noise-producing jet structure with high accuracy and to map them in time and frequency. Finally, we show how the relationships found can be applied to volcanic scenarios by scaling the results of this study to infer the conditions at the source of short-lived explosive volcanic eruptions. These experiments serve as a starting point to empirically constrain the link between acoustic fingerprints of compressible starting gas jets (experimentally generated and repeatable) to acoustic signals measured during impulsive volcanic explosions.

## Methods

### Shock tube discharge

The jets are generated with the vertical shock tube apparatus originally developed by Alidibirov and Dingwell^30^ and used by Cigala et al.^23^ to investigate the dynamics of particle-laden starting jets at volcanic conditions. The shock tube (made of Nimonic 105 alloy) allows for high P–T conditions (up to 100 MPa and 850 °C). The shock tube has an internal diameter of 28 mm (D_st_) and is 60 or 240 mm high (L). To reduce the length of the tube from 240 to 60 mm and thus the internal volume, a full cylinder of Teflon of 180 mm is being fixed at the bottom of the shock tube. The internal volume of 0.032 or 0.127 l, respectively, is being pressurized by Argon gas. The shock tube system is sealed from ambient conditions via one tailored metal disk. Depending on the desired pressure ratio, a disk of aluminum foil, copper or iron was chosen (in order of increasing stability). The combination of the type of metal, its thickness and imprint depth of a ring and a cross is controlling the absolute pressure differential that a disk can withstand. Empirically constrained disks with reliable opening pattern and stability have been chosen. The disk bursts at the desired overpressure in four triangle-shaped segments (less regular for the aluminum disk only) that are being bent upwards by 90° and end along the shock tube walls, generating some internal diameter variation (between 26 and 28 mm). At the shock tube exit, three nozzle geometries have been deployed: a cylindrical and two funnel-shaped nozzles. The geometries are parametrized by the exit-to-throat area ratio (A_e_/A^*^) = (1, 2.37, 4). In this study, all experiments took place at chamber temperature. Four overpressure scenarios (3, 4, 50 and 80 bar, respectively) have been investigated, with each set of experimental conditions having been repeated two times, resulting in pressure scenarios of 3.2 ± 0.1, 3.6 ± 0.3, 47.5 ± 2.6 and 78.4 ± 4.7 bar.

### Acoustic data acquisition system

The half-inch microphone used is manufactured by PCB with a frequency range of 3.75–20,000 Hz. The sampling rate is 102 kHz. We have analyzed the jet noise recorded in the near field with one microphone located at the shock tube exit plane at 0.35 m from the shock tube axis (see Fig. 2 for reference). The measurements have been triggered manually, taking into consideration the release of the pressure in the shock tube.

### Anechoic chamber

We have performed the measurements in the anechoic chamber facility of the Technische Universität Berlin. It has a free volume of 1,070 m^3^ and a walkable area of 126 m^2^. The certified lower frequency limit of the anechoic chamber is 63 Hz. This means that signals with lower frequencies than the lower bound of the anechoic chamber will be affected by the acoustic properties of the anechoic chamber, and the results will not be trustworthy. We cannot make any statement for frequencies lower than the lower bound of the anechoic chamber.

### Wavelet analysis of acoustic signals

To locate the different noise sources in frequency and time and retrieve more information about the physical processes that led to these acoustic signals, we have performed a time–frequency analysis of the acoustic signals recorded. For the analysis, we chose the wavelet transform over the short-time Fourier transform because the former has a constant relative error in frequency space while the latter has a constant absolute error in frequency space^25^,this difference allows a suitable resolution over the whole frequency range being able to identify better the noise sources in time–frequency. After testing different waveforms, we have performed the wavelet transformation using the complex Morlet wavelet and we have chosen the bandwidth parameter f_b_ = 1.5 and a center frequency of f_c_ = 1. The start and end frequencies are chosen to match the nominal frequency range of the microphones. With this setup, we achieved a relative error in the frequency space of about 4%. We used a logarithmic distribution of the frequencies during the computation of the wavelet coefficients for better visualization of the spectrograms and an easier interpretation of the results.

## Abbreviations

A_e_: Exit area
A^*^: Throat area
c_0_: Speed of sound in the reservoir at start
c_e_: Speed of sound at exit conditions
c_j_: Speed of sound at fully expanded conditions
D_e_: Exit diameter
D_j_: Diameter of the jet for fully expanded conditions
D_st_: Diameter of the shock tube
D_vr_: Diameter of the vortex ring
dt_s-sl-vr_: Time delay between the compression wave and the shock-shear layer-vortex ring interaction
f_BBSN_: Frequency of the broad-band shock noise
f_vr_: Frequency of the vortex ring noise
f_b_: Bandwidth parameter of the complex Morlet transform
f_c_: Center frequency of the complex Morlet transform
L: Reservoir length
L_s_: Shock cell spacing
M: Mach number
M_d_: Exit Mach number at the adapted conditions for the specific geometry
M_j_: Mach number at fully expanded conditions
Re: Reynolds number
R_g_: Specific gas constant
p_r_: Gas pressure in the reservoir at start
p_∞_: Gas pressure at unbounded chamber conditions
t: Time
t*: Characteristic time
T_j_: Temperature at fully expanded conditions
T_r_: Temperature in the reservoir at start
T_∞_: Temperature at unbounded chamber conditions
u_j_: Flow velocity at fully expanded conditions
u_KH_: Convection velocity of Kelvin–Helmholtz instability
u_vr_: Propagation velocity of the vortex ring
π: Pi
γ: Isentropic exponent
μ: Ratio core to vortex ring diameter
σ_1_: First root of the zero-order Bessel function
